# The differential mediating effects of pain and depression on the physical and mental dimension of quality of life in Hong Kong Chinese adults

**DOI:** 10.1186/1477-7525-8-1

**Published:** 2010-01-07

**Authors:** Wing S Wong, Simon TM Chan, Vivian BK Fung, Richard Fielding

**Affiliations:** 1 Department of Applied Social Studies, City University of Hong Kong, Kowloon Tong, Hong Kong; 2 Department of Social Work, Hong Kong Baptist University, Hong Kong; 3 Department of Community Medicine, The University of Hong Kong, Pokfulam, Hong Kong

## Abstract

**Objective:**

The impact of pain and depression on health-related quality of life (QoL) is widely investigated, yet the pain-depression interactions on QoL remain unclear. This study aims to examine the pain-depression-QoL mediation link.

**Methods:**

Pain severity were assessed in a sample of Chinese professional teachers (n = 385). The subjects were also assessed on depressive symptoms and QoL. Regression models were fitted to evaluate the pain-depression-QoL relationships.

**Results:**

About 44% of the sample had 3-5 painful areas in the past 3 months. Shoulder pain (60%) and headache (53%) were common painful areas. The results of regression analyses showed that pain mediated the effects of depression on the mental aspect of QoL (standardized *β *= -0.111; Sobel test: z = -3.124, *p *< 0.005) whereas depression mediated the effects of pain on the physical aspect of QoL (standardized *β *= -0.026; Sobel test: z = -4.045, *p *< 0.001).

**Conclusions:**

Our study offered tentative evidence that pain and depression impacted differently on the mental and physical aspect of QoL. As these findings were based on a Chinese teacher sample, future studies should employ more representative samples across cultures to verify the present data.

## Introduction

Chronic pain and depression are often co-morbid. The prevalence of depression among pain patients ranges from 10% [1] to 100% [2], whereas about 30% [3] to 100% [4] of depressed patients report pain. The nature and mechanism of the pain-depression association has been widely investigated [5], yet remain largely inconclusive. Research on the causal direction(s) of the pain-depression relationship has focused on three major lines of investigation [6]. First, depression as a precursor for pain, sensitizing a person to experience pain [7]. Second, depression as a result of pain due to the sustained reduction in physical and social activities [8]. Lastly, depression and pain share the same or similar underlying biophysiological mechanisms [9].

The impact of pain and depression on health-related quality of life (QoL) has been well documented. Pain impacts different aspects of QoL and impairs general health perceptions among cancer patients [10]. Pain also impairs mental and physical functioning and generates severe anxiety [11]. Depressed individuals generally report poorer QoL [12]. Depression predicted QoL among bereaved adults [13] and in patients with cancer [14]. Both pain and depression independently predicted QoL in Chinese cancer patients [15]. Despite these links between QoL, pain and depression, clarification of any pain-depression interactions impacting QoL is lacking. Specifically, the extent to which pain exerts differential effects on QoL with different domains was unclear. We therefore explored the relationships between pain, depression, and QoL, considering the mediating effects of both pain and depression on two core dimensions of QoL, physical and mental.

## Methods

Following IRB approval, questionnaires were sent to 14 primary schools which were randomly selected from the New Territories district in Hong Kong. A total of 385 (response rate: 72%) professional teachers completed the questionnaires. About 78% were females and over half fell into the age group of 21-30 (31.7%) or 31-40 (38.1%) years. About 40% were Christians whereas 52% were married.

### Pain severity

Pain severity was first identified by affirmative answer to a question, "Are you currently troubled by physical pain for ≥ 3 months?" Subjects answering yes to the question were then assessed using the Chronic Pain Grade (CPG) questionnaire [16], a seven-item instrument assessing severity in three dimensions: persistence, intensity and disability. The three intensity items ask respondents to rate their current, average and worst pain intensity on 0 - 10 Numerical Rating Scales (NRS) (0 = "No pain at all"; 10 = "Pain as bad as could be"). A Characteristic Pain Intensity Score (score range: 0-100) is derived by averaging the responses to the intensity items and multiplying this by 10. Three CPG items assess pain interference with (1) daily activities, (2) social activities, and (3) working ability using 0 - 10 NRSs (0 = "No interference/change"; 10 = "Unable to carry on activities/extreme change"). The CPG Disability Score (score range: 0-100) is derived by multiplying the average of the three interference items by 10. Persistence is assessed in the original CPG by asking the respondent to indicate the number of days out of the past six months days that he or she was disabled by pain (although we modified this to "the past three months" because chronic pain is now defined as pain that persists for at least three months^24^). The Disability Score and the number of disability days are recoded into 5-point scales (Disability Score: 0 = "0-29", 1 = "30-49", 2 = "50-69", 3 = "70 or above"; Disability Days: 0 = "0-6 days", 1 = "7-14 days", 2 = "15-30 days", 3 = "31 days or above") and summed, yielding "Disability Points". Based on the Pain Intensity Score and Disability Points, CPG classifies chronic pain into five hierarchical grades: Grade Zero (pain free), Grade I (low disability-low intensity), Grade II (low disability-high intensity), Grade III (high disability-moderately limiting) and Grade IV (high disability-severely limiting). Previous reports indicated that CPG is a valid and reliable instrument [17]. The Chinese version of CPG also demonstrated good psychometric properties in a Chinese community sample [18].

### Depression

The 7-item depression subscale of the Hospital Anxiety and Depression Scale (HADS-D) [19] was employed to evaluate depressive symptoms of the respondents. The HADS-D is scored between 0 and 21, with higher scores indicating greater levels of depressive symptoms. The Chinese version has good psychometrics [20]. A cut-off score of 8 was recommended for HADS-D for both the Western and Chinese population [19,20].

### Quality of life

Respondents also answered the Medical Outcomes Study 12-item Short-Form Health Survey (SF12) [21]. The 12 questions are summarized into a physical component (SF12-PCS) score and a mental component score (SF12-MCS). The SF12 has been translated into Chinese and validated in Hong Kong [22].

### Statistical Analysis

Descriptive statistics assessed pain characteristics, depression, and QoL scores of the sample. Regression models were used to investigate the pain-depression-QoL mediation chain. Separate models were fitted to SF12-MCS and SF12-PCS in examining QoL as an outcome variable. For pain to be a mediator of depression and QoL, four criteria as proposed by Baron and Kenny [23] need to be met: (1) depression should significantly predict pain, (2) pain should significantly predict QoL, (3) depression should significantly predict QoL, and (4) controlling for pain, the relationship between depression and QoL should be reduced or no longer significant. Perfect mediation is established if the association between depression and QoL is reduced to zero. The Sobel test [24] determined whether pain carried the influence of depression to QoL. These criteria were also applied to test the mediating effect of depression. A series of four regression models were used to individually test each of these three-variable mediation chains. The results of separate regression analyses showed none of the socio-demographic variables predicted QoL (all *p *> 0.05); they were therefore dropped as covariates from subsequent regression models. In all regression analyses, the pain variable was indexed by the CPG classification as it takes into account both pain intensity and pain disability. All data analyses were performed using SPSS version 15.0.

## Results

### Prevalence of pain, CPG classification, and means scores of depression and QoL measures

Only 8% of the sample reported no pain symptom in the past 3 months (Table 1). Those with pain symptoms (92%) experienced an average of 3.81 painful areas (SD = 2.53) with 44% reporting 3-5 painful areas. Of the symptomatic subjects, 20% met the classification of Grade III or above. The proportions of those being classified as Grade Zero, Grade I, and Grade II were 0%, 31.1%, and 45.3% respectively. The mean scores of HADS-D, SF12-MCS, and SF12-PCS were 7.63 (SD = 3.87), 35.72 (SD = 5.75), 35.80 (SD = 9.28) respectively.

**Table 1:** Pain characteristics and means of depression and QoL measures

Pain measures	n (%)
Number of pain areas^a^, M (SD)	3.81 (2.53)
0	30 (8)
1-2	95 (25)
3-5	164 (44)
6-9	75 (20)
10+	10 (3)
Pain locations^b^	
Shoulder	224 (60)
Head	199 (53)
Neck	176 (47)
Leg	166 (44)
Back	149 (40)
Hand	95 (25)
Stomach	93 (25)
Joint	75 (20)
Menstrual	73 (20)
Abdomen	63 (17)
Tooth	51 (14)
Chest	48 (13)
Others	12 (3)
Chronic Pain Grade classification^b^	
Grade 0	0
Grade I	107 (31.1)
Grade II	156 (45.3)
Grade III	65 (18.9)
Grade IV	4 (1.2)
Pain Intensity, M (SD)	56.41 (16.95)
Depression^c^, M (SD)	7.63 (3.87)
Quality of life, M (SD)	
SF12-MCS	35.72 (5.75)
SF12-PCS	35.80 (9.28)

Note: SF12: Medical Outcomes Study 12-item Short-Form Health Survey; MCS: Mental Component Score; PHS: Physical Component Score; M: Mean; SD: Standard deviation.

^a ^A total of 11 cases with missing data on the number of pain areas item.

^b^Only respondents with at least one pain location (n = 344) were included in the analyses. Twelve respondents with pain symptoms had missing data on the CPG items.

^c^Indexed by Hospital Anxiety and Depression Scale Depression subscale. Higher scores indicate greater psychological distress.

### Mediation in the pain-depression-QoL relationships

**Figure 1:**
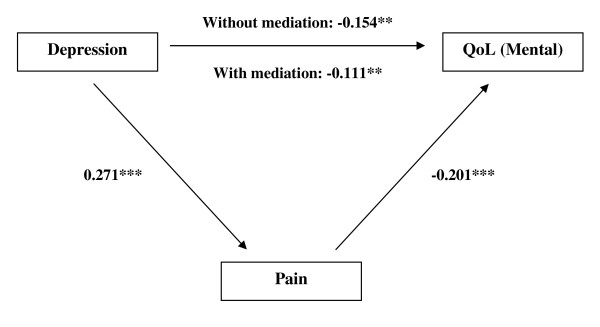
**Standardized beta coefficient in pain partially mediated pathway from depression to QoL (mental)**. ** *p *< 0.005; *** *p *< 0.001.

The results of Model 1 (Table 2) showed a significant inverse relationship between depression scores and mental QoL (*β *= -0.154, *p *< 0.05), whereas depression was positively related to pain (*β *= 0.271, *p *< 0.001). Pain was significantly and inversely related to QoL (*β *= -0.201, *p *< 0.001). When mediation was controlled, depression remained inversely associated with QoL (*β *= -0.111, *p *< 0.005), demonstrating a partial mediation effect of pain between depression and QoL. Sobel's test indicated pain's role as a mediator the depression-QoL relationship (*z *= -3.124, *p *< 0.005) (Figure 1).

**Table 2:** Regression models testing the Pain-Depression-QoL mediation chain^a^

Model	Std β	SE	95% CI	P value
**Model 1: Pain mediates the Depression-QoL (Mental)^b ^link**				
Depression (Predictor) → QoL (Outcome)	-0.154	0.078	-0.381, -0.074	0.004
Pain (Mediator) → QoL (Outcome)	-0.201	0.119	-0.689, -0.222	<0.001
Depression (Predictor) → Pain (Mediator)	0.271	0.033	0.113, 0.243	<0.001
Depression (Predictor) → QoL (Outcome)|Pain (Mediator)^c^	-0.111	0.081	-0.628, -0.143	0.002
Sobel test	**z = -3.124**		**P = 0.002**	
				
**Model 2: Pain mediates the Depression-QoL (Physical)^d ^link**				
Depression (Predictor) → QoL (Outcome)	-0.311	0.121	-0.982, -0.504	<0.001
Pain (Mediator) → QoL (Outcome)	-0.106	0.195	-0.769, -0.004	0.048
Depression (Predictor) → QoL (Outcome)|Pain (Mediator)^c^	-0.304	0.127	-0.981, -0.480	<0.001
Sobel test	**z = -1.929**		**P = 0.054**	
				
**Model 3: Depression mediates the Pain-QoL (Mental)^b ^link**				
Pain (Predictor) → QoL (Outcome)|Depression (Mediator)^e^	-0.170	0.123	-0.324, -0.006	0.042
Sobel test	**z = -1.853**		**P = 0.064**	
				
**Model 4: Depression mediates the Pain-QoL (Physical)^d ^link**				
Pain (Predictor) → QoL (Outcome)|Depression (Mediator)^e^	-0.026	0.195	-0.479, 0.286	0.620
Sobel test	**z = -4.045**		**P < 0.001**	

Note: QoL: Quality of life; Std *β*: Standardized beta coefficient; SE: Standard error; CI: Confidence interval.

^a^Four separate regression models were generated to test the pain-depression-QoL mediation chain.

^b^QoL was indexed by SF12-MCS.

^c^Pain, as mediator, was controlled in the regression equation.

^d ^QoL was indexed by SF12-PCS.

^e^Depression, as mediator, was controlled in the regression equation.

In Model 2, depression was inversely associated with physical QoL (*β *= -0.311, *p *< 0.001) and pain was inversely associated with QoL (*β *= -0.106, *p *< 0.05). After controlling for pain, depression remained significantly associated with QoL (*β *= -0.304, *p *< 0.001). The result of Sobel test however suggested the reduction in standardized beta coefficients after controlling for mediation was not significant (z = -1.929, *p *> 0.05)

Results of Model 3 indicated that after controlling for depression, pain significantly associated with mental QoL (*β *= -0.170, *p *< 0.05). Although the standardized beta coefficients were reduced after controlling for mediation, the reduction was not statistically significant as suggested by the Sobel test (z = -1.853, *p *> 0.05).

**Figure 2:**
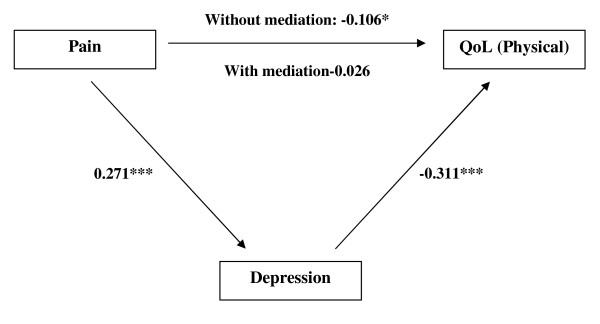
**Standardized beta coefficient in depression partially mediated pathway from pain to QoL (physical)**. * *p *< 0.05; *** *p *< 0.001.

In Model 4, after controlling for depression, pain no longer significantly associated with physical QoL (*β *= -0.026, *p *> 0.05). The result of Sobel test offered further evidence for the partial mediating effect of depression to the pain-QoL association (*z *= -4.045, *p *< 0.001) (Figure 2).

## Discussion

We know of no other prior studies that evaluated the pain-depression-QoL mediation chain by testing the differential effects on the physical and mental dimension of QoL independently. Regression analyses showed that pain and depression impacted differently on the mental and physical aspect of QoL. Pain significantly mediated the depression-QoL link for the mental aspect (Model 1) whereas depression significantly mediated the pain-QoL link for the physical aspect (Model 4). When pain was the mediator, it accentuated the negative effects of depression on mental functioning. The indirect effect of pain on the depression-QoL pathway was 0.043, suggesting that about 28% of the effect of depression on the mental aspect of QoL went through the mediator of pain, and over 72% of the effect was direct. When depression was the mediator, depressive symptoms accentuated the negative effects of pain on physical QoL. The indirect effect of depression on the pain-QoL link pathway was 0.08, indicating that about 75% of the effect of pain on QoL impacted through the mediator of depression, and 25% of the effect was direct. Statistically, the mediation of depression (Model 4: 75%) exerted the strongest effect (cf. Model 1: 28%). These findings offer insights to the current understanding on the pain-depression relationship that whether pain and depression be a predictor or mediator in impacting QoL is dependant on the specific aspect of QoL in question.

The high prevalence of pain symptoms (92%) might be explained by the ubiquitous use of computers in the teaching profession. Yet, further investigation is needed to confirm the current prevalence estimate of chronic pain among professional teachers. It should be noted that among the symptomatic subjects, 28.5% of them were classified as Grade Zero, suggesting that pain did not lead to disability among these subjects. Research has documented a higher tendency for somatization in Chinese culture [25]. However, as we did not assess pain etiology or somatization, we cannot determine from the present data whether somatization contributed to the high prevalence of pain.

Despite the significant findings from this exploratory study, the relationship between pain, depression, and QoL should be considered tentative. While the present study assessed pain severity, future investigations should explore how different dimensions of pain (e.g., pain location and number of pain areas) impact depression and QoL. As other factors may also be involved in the mediation chain, future attempts should also explore the possible range of interaction between variables. Also, the extent to which causes of pain symptoms influence the relationship between pain and physical QoL remain unclear; this issue should be addressed in future research. The cross-sectional design of this study did not allow us to infer causality. Cautions should be exercised when interpreting and generalizing the current findings in other populations as the present sample consisted of mainly Chinese female (78.2%) teachers. Previous studies show that the experience of pain varies across cultures. Replication of the present findings in other cultures is therefore warranted [26,27]. Even within the Chinese population, future studies should validate the present finding using a more representative sample with diverse socio-economic background.

## Competing interests

The authors declare that they have no competing interests.

## Authors' contributions

WSW, STMC and RF designed the questionnaire. WSW and VBKF performed statistical analysis. WSW drafted the manuscript; RF participated in editing the manuscript.

All authors read and approved the final manuscript.

## References

[B1] Piloswky I Chapman CR Bonica JJ Pain, depression, and illness behavior in a pain clinic population Pain 1977 4 183 192 10.1016/0304-3959(77)90132-4 600541

[B2] Turkington RW Depression masquerading as a diabetic neuropathy JAMA 1980 243 1147 1150 10.1001/jama.243.11.1147 6987418

[B3] Gallemore JL Wilson WP The complaint of pain in the clinical setting of affective disorders Southern Medical Journal 1969 62 551 555 5782183 10.1097/00007611-196905000-00012

[B4] Ward NG Bloom VL Friedel RO The effectiveness of tricyclic antidepressants it the treatment of coexisting pain and depression Pain 1979 7 331 341 10.1016/0304-3959(79)90089-7 530739

[B5] Brown GK A causal analysis of chronic pain and depression Journal of Abnormal Psychology 1990 99 127 137 10.1037/0021-843X.99.2.127 2348006

[B6] Robinson ME Riley JL Gatchel RJ, Turk DC The role of emotion in pain Psychosocial factors in pain: Clinical perspective 1999 New York: Guilford 74 88

[B7] Blumer D Heilbronn M Chronic pain as a variant of depressive disease: The pain-prone disorder Journal of Nervous and Mental Disease 1982 170 381 406 10.1097/00005053-198207000-00001 7086394

[B8] Hendler N Depression caused by chronic pain Journal of Clinical Psychiatry 1984 45 30 36 6698950

[B9] Magni G On the relationship between chronic pain and depression when there is no organic lesion Pain 1987 31 1 21 10.1016/0304-3959(87)90002-9 3320879

[B10] Anie KA Steptoe A Bevan DH Sickle cell disease: Pain, coping and quality of life in a study of adults in the UK British Journal of Health Psychology 2002 7 331 334 10.1348/135910702760213715 12614504

[B11] Wang XS Cleeland CS Mendoza TR Engstrom MC Liu S Xu G Hao X Wang Y Ren XS The effects of pain severity on health-related quality of life: a study of Chinese cancer patients Cancer 1999 86 9 1848 1855 10.1002/(SICI)1097-0142(19991101)86:9<1848::AID-CNCR29>3.0.CO;2-M 10547560

[B12] Moses T Leuchter AF Cook I Abrams M Does the clinical course of depression determine improvement in symptoms and quality of life? The Journal of nervous and mental disease 2006 194 4 241 248 10.1097/01.nmd.0000207358.15230.80 16614544

[B13] Boelen PA Prigerson HG The influence of symptoms of prolonged grief disorder, depression, and anxiety on quality of life among bereaved adults: A prospective study European archives of psychiatry and clinical neuroscience 2007 257 8 444 452 10.1007/s00406-007-0744-0 17629728

[B14] Skarstein J Aass N Fossa SD Skovlund E Dahl AA Anxiety and depression in cancer patients: relation between the Hospital Anxiety and Depression Scale and the European Organization for Research and Treatment of Cancer Core Quality of Life Questionnaire Journal of psychosomatic research 2000 49 1 27 34 10.1016/S0022-3999(00)00080-5 11053601

[B15] Wong WS Fielding R Eating ability predicts subsequent quality of life in Chinese patients with breast, liver, lung, or nasopharyngeal carcinoma: A longitudinal analysis Acta Oncologica 2008 47 1 71 80 10.1080/02841860701441814 18097779

[B16] Von Korff M Dworkin SF Le Resche L Graded chronic pain status: an epidemiologic evaluation Pain 1990 40 3 279 291 10.1016/0304-3959(90)91125-3 2326094

[B17] Elliott AM Smith BH Smith WC Chambers WA Changes in chronic pain severity over time: the Chronic Pain Grade as a valid measure Pain 2000 88 3 303 308 10.1016/S0304-3959(00)00337-7 11068118

[B18] Fielding R Wong WS The prevalence of chronic pain, fatigue, and insomnia in the general population of Hong Kong. Final report to the Health, Welfare and Food Bureau, Government of the Hong Kong Special Administrative Region, China 2008 Hong Kong: School of Public Health, the University of Hong Kong

[B19] Zigmond AS Snaith RP The hospital anxiety and depression scale Acta Psychiatr Scand 1983 67 6 361 370 10.1111/j.1600-0447.1983.tb09716.x 6880820

[B20] Leung CM Ho S Kan CS Hung CH Chen CN Evaluation of the Chinese version of the Hospital Anxiety and Depression Scale. A cross-cultural perspective Int J Psychosom 1993 40 1-4 29 34 8070982

[B21] Ware JE SD-36 Health Survey Manual and Interpretation Guide 1993 Boston: Nomrod Press

[B22] Lam CL Tse EY Gandek B Is the standard SF-12 health survey valid and equivalent for a Chinese population? Qual Life Res 2005 14 2 539 547 10.1007/s11136-004-0704-3 15892443

[B23] Baron RM Kenny DA The moderator-mediator variable distinction in social psychological research: Conceptual, strategic, and statistical considerations Journal of Personality and Social Psychology 1986 6 1173 1182 10.1037/0022-3514.51.6.1173 3806354

[B24] MacKinnon DP Lockwood CM Hoffman JM West SG Sheets V A comparison of methods to test mediation and other intervening variable effects Psychological Methods 2002 7 83 104 10.1037/1082-989X.7.1.83 11928892 PMC2819363

[B25] Mak WW Zane NW The phenomenon of somatization among community Chinese Americans Soc Psychiatry Psychiatr Epidemiol 2004 39 12 967 974 10.1007/s00127-004-0827-4 15583904

[B26] Sheffield D Biles PL Orom H Maixner W Sheps DS Race and sex differences in cutaneous pain perception Psychosomatic Medicine 2000 62 517 523 10949097 10.1097/00006842-200007000-00010

[B27] Edwards RR Doleys DM Fillingim RB Lowery D Ethnic differences in pain tolerance: Clinical implications in a chronic pain population Psychosomatic Medicine 2001 63 316 323 11292281 10.1097/00006842-200103000-00018

